# Discontinuous nature of the repulsive-to-attractive colloidal glass transition

**DOI:** 10.1038/srep22725

**Published:** 2016-03-04

**Authors:** T. van de Laar, R. Higler, K. Schroën, J. Sprakel

**Affiliations:** 1 Physical Chemistry and Soft Matter, Wageningen University, Wageningen, The Netherlands; 2 Laboratory of Food Process Engineering, Wageningen University, Wageningen, The Netherlands

## Abstract

In purely repulsive colloidal systems a glass transition can be reached by increasing the particle volume fraction beyond a certain threshold. The resulting glassy state is governed by configurational cages which confine particles and restrict their motion. A colloidal glass may also be formed by inducing attractive interactions between the particles. When attraction is turned on in a repulsive colloidal glass a re-entrant solidification ensues. Initially, the repulsive glass melts as free volume in the system increases. As the attraction strength is increased further, this weakened configurational glass gives way to an attractive glass in which motion is hindered by the formation of physical bonds between neighboring particles. In this paper, we study the transition from repulsive-to-attractive glasses using three-dimensional imaging at the single-particle level. We show how the onset of cage weakening and bond formation is signalled by subtle changes in local structure. We then demonstrate the discontinuous nature of the solid-solid transition, which is marked by a critical onset at a threshold bonding energy. Finally, we highlight how the interplay between bonding and caging leads to complex and heterogeneous dynamics at the microscale.

The vitrification of colloidal hard spheres is accompanied by a rapid rise in structural relaxation time when the particle volume fraction *ϕ* is increased in proximity of the glass transition at *ϕ*_*g*_ ≈ 0.58. The reduction in free volume at these densities leads to the emergence of configurational cages in which particle motion is restricted by neighbouring particles^1^,^2^,^3^. The introduction of short-ranged attractive interactions to a hard sphere suspension in the supercooled regime leads to a departure from this well-established caging picture^4^,^5^,^6^. Low attraction strengths, *U* ≈ *k*_*B*_*T*, give rise to weak clustering which increases the local free volume and melts the glass^4^,^7^. Upon increasing the strength of attractive interactions, physical bonds between neighboring particles form, whose lifetime grows with attraction strength^8^. This causes a re-entrance into a bonding-dominated glassy state whose properties are distinctly different from the repulsive glass^9^,^10^.

In dilute suspensions of colloids, short-ranged attractions lead to phase separation and kinetic arrest. This result in the formation of a highly heterogeneous solid state; the colloidal gel^10^,^11^,^12^. It has been proposed that the gel line in the (*U*, *ϕ*)-plane of the suspension phase diagram extends to the supercooled regime and causes the re-entrant transition to an attractive glass^4^,^6^,^7^,^13^. Nonetheless, several key questions about the nature of this solid-solid transition between two very different amorphous states remain unanswered.

For example, what is the nature of dynamical arrest at the onset of the attractive glass transition. In repulsive systems arrest occurs by the formation of confining cages which slow down particle self-diffusion in proximity of the glass transition *ϕ*_*g*_. Even though the decrease of particle mobility is steep, the transition is continuous as the system remains ergodic up to, and beyond, *ϕ*_*g*_^14^. By contrast, the gel point in dilute suspensions of attractive colloids is characterised by both structural and dynamical signs of a critical percolation transition^11^,^12^,^15^. Finally, in colloids which bond with a well-defined valency, yet another scenario is reported, where the glass transition exhibits Arrhenius behavior with a relaxation time *τ* ∝ exp(*U*/*k*_*B*_*T*) and no critical onset^16^. This raises the question how a dense suspension of paricles undergoes a transition to an attractive glass, where bonding forces rule, but remnants of configurational cages persist. Moreover, our understanding of how interactions influence the spatial homogeneity of local dynamics in three-dimensional glasses is incomplete. For purely repulsive system the length scale associated with dynamical heterogeneity is known to grow strongly upon increasing the particle volume fraction^17^. For two-dimensional glasses, experiments have shown a transition from string-like cooperative motion for repulsive particles, to condensed islands of larger mobility at moderate attractions^8^. However, it was recently established that there are distinct differences in the glass transition in two and in three dimensions^18^. It thus remains a challenge to establish the transitions in global and local dynamics occurring during the attractive glass transition in three dimensions.

In this paper we use three-dimensional confocal fluorescence imaging to explore the nature of the transition from repulsive to attractive colloidal glasses upon introducing weak bonding interactions. We show how small changes in local structure signal the onset of bond formation. This results in a critical onset for the attractive glass. At the edges of the intermediate supercooled liquid state, we find a distinct discontinuity in the length scale associated with heterogeneous dynamics. Our results evidence the proposed connection between gels and attractive glasses^10^,^13^, and shed new light on this transition between two disordered solids governed by different microscopic physics.

## Results and Discussion

We study a glass of colloidal hard spheres in a solvent mixture that closely matches the density of the particles; this allows us to study equilibrium bulk behavior in absence of gravitational stresses. In this solvent mixture, also the refractive index of the fluorescent colloids is matched, enabling us to look deep inside the suspension with confocal fluorescence microscopy. The suspensions consists of a bidisperse mixture, with particles of *a*_*A*_ = 0.89 and *a*_*B*_ = 1.23 *μm*, to avoid crystallisation even after prolonged equilibration. We note that we do not observe decoupling between the diffusivity of the two sizes for this size ratio^19^. The sample bidispersity completely prevents local crystalline order.

To introduce attractive interactions we add polystyrene microgels to the suspension (*a*_*μg*_ = 21 nm), which induce a depletion attraction between the larger colloidal hard spheres^20^. The depth of the attractive minimum due to the depletion of the larger hard spheres by the smaller microgels is approximated as 
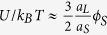
20, where 
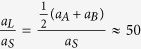
 is the size ratio of hard spheres to depletant and *ϕ*_*S*_ the volume fraction of polystyrene microgels, which we determine by capillary viscosimetry.

### Structure

**Figure 1:**
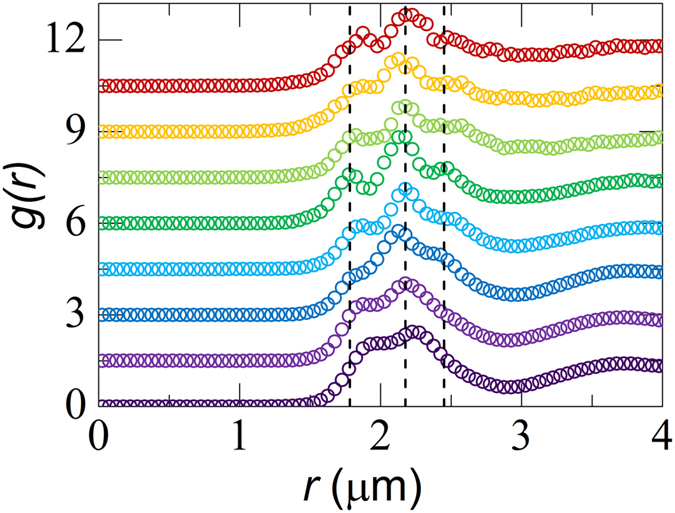
Pair correlation functions as a function of attraction strength, with (bottom to top): *U*/*k*_*B*_*T* = 0, 0.8, 2.5, 3.1, 4.6, 5.2, 6.7 and 8.3. Curves have been offset vertically for clarity. Dotted lines indicate bonding distances between *AA*, *AB* and *BB* pairs in the bidisperse glass.

The purely repulsive glass exhibits a liquid-like pair correlation function *g*(*r*) that is devoid of any signs of long- or medium-ranged order (Fig. 1). This illustrates how the binary size ratio we use here thus frustrates the systems sufficiently that ordering is prevented completely. The global shape of the pair correlation function is unaffected by increasing the interparticle attraction energy (Fig. 1). However a subtle change in the nearest-neighbor peak is observed when interparticle attractions become significant. As *U* increases, bonds between neighboring particles form, which gives rise to a distinct fine-structure in the first peak of *g*(*r*). Since the sample is composed of two distinct populations, three sub-peaks emerge indicative of *AA*, *AB* and *BB* bonds (dotted lines Fig. 1). Thus, while weak attraction does not change the global structure of the glass, subtle changes occur at the nearest-neighbor level.

### Global dynamics

**Figure 2:**
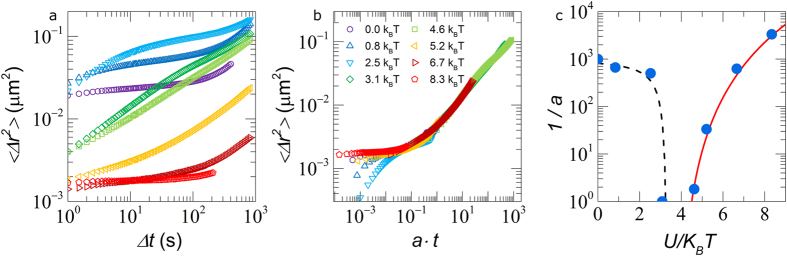
(**a**) Mean-squared displacements 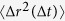 for all attraction strengths. (**b**) The same mean-squared displacements superposed onto a single master curve, symbols and colors are similar for both figures. (**c**) Shows shift factor 1/*a* as a function of *U*: solid line is a fit to the data with 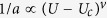, dotted line is a guide to the eye.

To explore how these changes in local structure affect the global dynamics of the glass we calculate the ensemble-averaged mean-squared displacement 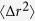. For the repulsive glass we find the characteristic short-time cage rattling motion leading into a caging plateau and the subsequent upturn towards long-time diffusion at longer times due to cage breaking (Fig. 2a). For weak attractions, *U* < 3 *k*_*B*_*T*, 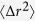 shifts upwards signaling the gradual “melting” of the glass. Upon crossing a threshold attraction strength *U* ≈ 3 *k*_*B*_*T*, corresponding to where bonding peaks emerged in the *g*(*r*), 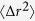 strongly decreases with increasing attraction strength as the system enters the attractive glassy state (Fig. 2a). This re-entrance was observed previously in both hard spheres^7^,^8^,^21^ and microgels^4^.

The structural relaxation time cannot be easily extracted directly from confocal microscopy experiments as we only have access to a limited range of lag times. We therefore rescale the mean-square displacements to a master curve, in analogy to the time-temperature superposition principle used for the rheology of molecular and polymeric glass formers^22^. We find a good collapse of the mean-squared displacements for time scales which are associated with cage- or bond-breaking and translational diffusion (Fig. 2b). This suggests a universal shape of the mean-squared displacements for these processes, with an observation window depending only on the ratio of experimental time versus the characteristic time scale for a particle to escape its neighbors. However, the rescaling does not collapse the short time dynamics. In this regime particles perform vibrations within a repulsive cage, or confined by attractive bonds; these dynamics depend mainly on the curvature of the confining potential rather than its absolute depth, and on the number of bonded neighbors.

We can now evaluate the attraction strength dependence of the shift factor *a* for the lag time axis, which relates to the structural relaxation time of the sample as *τ* ~ 1/*a* (Fig. 2c). The liquefaction of the repulsive glass, which results from a rise in free volume due to clustering, can be clearly observed at low *U*. The relaxation time decreases very strongly close to an attraction strength *U* ≈ 3.5 *k*_*B*_*T* where the glass melts, suggesting a steep dependence of particle diffusivity on free volume in proximity to the glass transition. This is also manifested by the fragility of the hard sphere glass transition along the *ϕ* axis^14^.

On the attractive side of the re-entrance *U* > 4 *k*_*B*_*T*, we see a strong rise in relaxation time, increasing by more than 3 orders of magnitude over a small range of *U* (Fig. 2c). In dilute suspensions, the dynamics and mechanics are established to exhibit a critical scaling at the onset of gelation. Also for the attractive glass transition we study, this scenario holds: our data is well described by the critical power-law 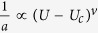, with *U*_*c*_ = 3.9 *k*_*B*_*T* the critical attraction strength for the onset of bonding-dominated arrest and *v* = 4 the critical exponent. This provides direct experimental evidence for the theoretical prediction that bonding-dominated arrests, whether it is gelation at low volume fractions or vitrification at high volume fractions, occurs by crossing a universal gel line that traverses the (*U*, *ϕ*)-plane of the suspension phase diagram.

### Local and heterogeneous dynamics

**Figure 3:**
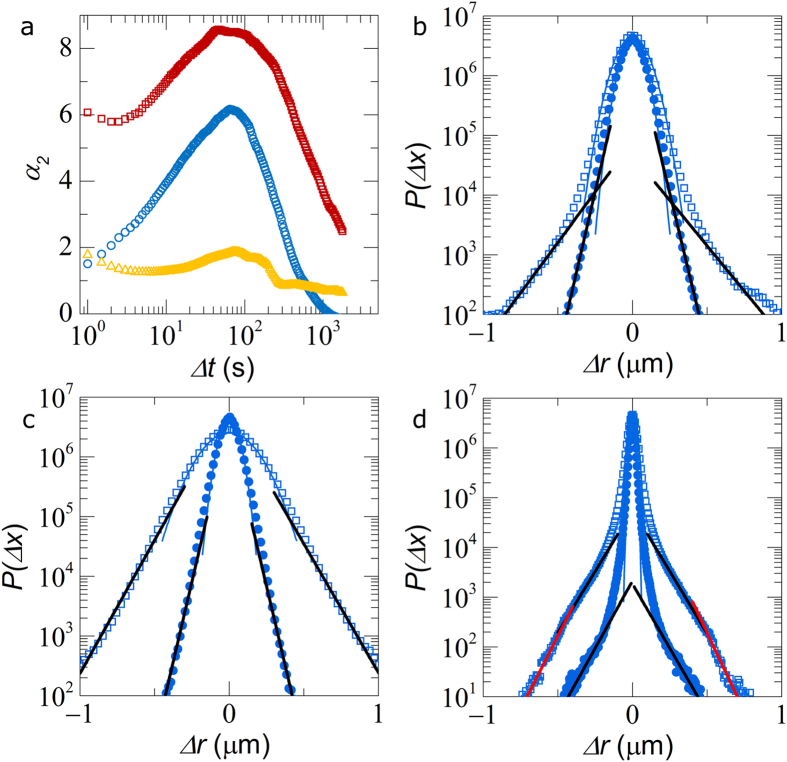
(**a**) Non-gaussian parameter *α*_2_(Δ*t*) for the repulsive glass (*U* = 0 *k*_*B*_*T*, circles), the supercooled liquid (*U* = 2.5 *K*_*B*_*T*, triangles) and attractive glass (*U* = 8.3 *k*_*B*_*T*, squares), (**b**–**d**) show corresponding particles displacement probabilities *P*(Δ*r*, Δ*t*) for Δ*t* = 0.5 s (circles) and *t* = t^*^ (squares). Lines are fits to Gaussian (blue) and exponential distributions (red and black) as discussed in the text.

Both the average global structure and dynamics display a distinct transition from a caging-dominated glass to a solid state governed by particle bond formation. We proceed by investigating dynamics at the local, single-particle, scale. We compute the Gaussianity of the particle displacements Δ*r* by means of the often used non-Gaussian parameter 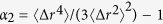. For the repulsive glass *U* = 0, we find strongly heterogeneous dynamics peaking at a characteristic time t^*^ ≈ 66 s (Fig. 3a). The corresponding displacement distributions *P*(Δ*r*) show largely Gaussian behavior for short times 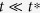 and a characteristic Gaussian distribution with exponential tails at t^*^ (Fig. 3b)^17^. As the glass melts, the dynamics become significantly more homogeneous, with *α*_2_ becoming much smaller over the entire range of time scales explored (Fig. 3a). Also the displacement distributions become more Gaussian, while exponential tails persist at the largest Δ*r* (Fig. 3c); indicating that due to the high *ϕ* in our experiments, the glass melts into the strongly supercooled regime, which still exhibits signatures of glassy dynamics.

In the attractive glass, the heterogeneous dynamics are most pronounced; *α*_2_ is finite and large across the entire spectrum, even for the shortest lag times we explore (Fig. 3a). Inspection of *P*(Δ*r*) shows two distinct populations at the shortest lag times *t* = 0.5 *s*. We observe a narrow Gaussian mode of small displacements, which we attribute to bond vibrations, and an exponential relaxation mode, due to intermittent debonding-induced local translation within configurational cages. These motions extend to somewhat larger distances, but do not exceed the typical size of the cages (Fig. 3d). As thermally-activated bond breaking is a Poisson process, even at the short times both bonded particles and those that have debonded and diffuse within a configurational cage must be present. At larger times *t* = t^*^, a second exponential relaxation mode becomes apparent, which results from particles which both debond and escape their configurational cages, which eventually leads to long-time translational motion. While the attractive glass transition is dominated by attractive bonds, the remnant of cages remain noticeable in the local glass dynamics, giving rise to complex relaxation dynamics. This is also reflected in the non-linear rheology of attractive glasses, which exhibit two distinct yielding processes associated with bond- and cage-breaking^9^,^10^.

**Figure 4:**
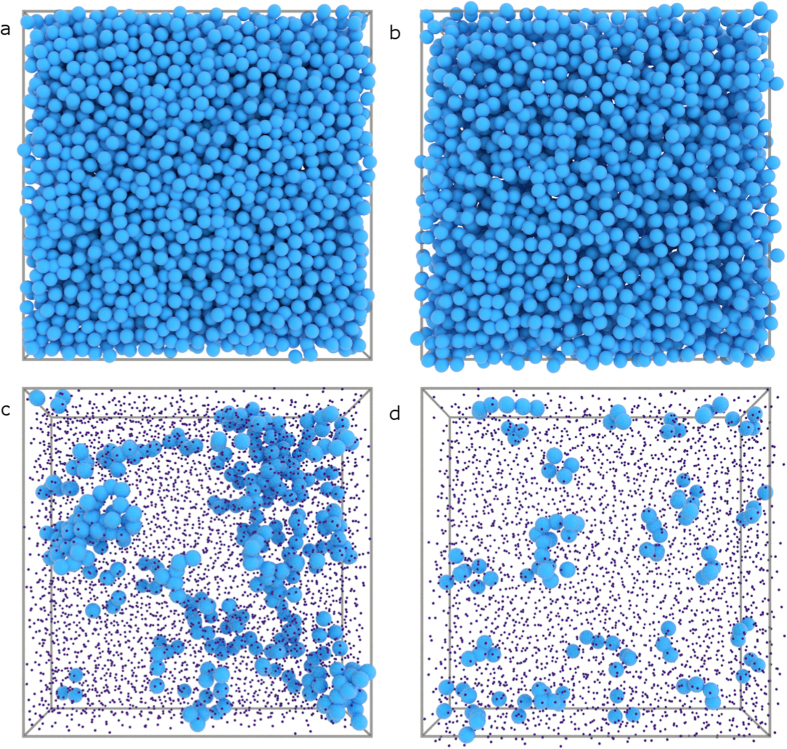
Computer-generated renderings of the experimental system for *U* = 0 *k*_*B*_*T* (**a**,**c**) and *U* = 8.3 *k*_*B*_*T* (**b**,**d**). Top row: all particles in the glass at approximately their real size, bottom row: highlighting the location of the fastest particles part of clusters with *N*_*c*_ > 2 in the glass, all others shown at reduced size for clarity.

**Figure 5:**
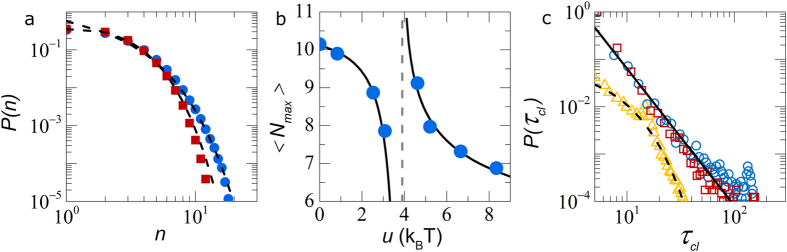
(**a**) Probability distributions of cluster sizes *P*(*N*) for the repulsive (circles) and attractive glass (squares), corresponding lines show fits to an exponential distribution. (**b**) Maximum average cluster size 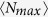 as a function of *U*. Solid lines indicate the discontinuity at *U*_*c*_ (dotted line). (**c**) Distribution of the lifetime of a particle in a connected cluster of fast particles *P*(*τ*_*cl*_), for repulsive (circles) and attractive glasses (squares) and the re-entrant liquid (triangles). Solid line is a powerlaw fit, dotted line an exponential distribution.

Finally, we evaluate the spatial homogeneity of these multiple populations which coexist within the glass adopting the approach described previously^17^. We first identify the particles with the largest displacements at t^*^. We select the 10% fastest particles, averaged over the entire length of the experimental observation. This means, that within each frame the number of fast particles can vary substantially. Clusters of these “fast” particles are determined based on proximity, allowing us to measure their size *N*. In all cases, a large amount of isolated fast particles, *N* = 1 are observed. To visualise the extent of clustering, we reconstruct our three-dimensional experimental data by showing “fast” particles part of a cluster with *N* > 2 at true size and all others at reduced size for visibility. While the purely repulsive glass displays several large, spatially extended clusters (Fig. 4c), particle dynamics appear more homogeneous for systems deep within the attractive glass regime (Fig. 4d). Indeed, the cluster size distributions *P*(*N*) show a subtle change in the probability of finding large clusters; with the repulsive glass being more prone to exhibiting spatial heterogeneity involving many particles (Fig. 4c). We note that, *P*(*N* > 2) at all attraction strengths, is well described by an exponential decay (dotted lines in Fig. 5a), indicative of a characteristic length scale associated with these heterogeneous dynamics.

As a measure for the spatial extent of clustering we measure the time-averaged size of the largest cluster in our field of view 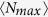. This clearly reveals a discontinuity, as predicted by mode-coupling theory, at the transition from repulsive to attractive glass^6^. The characteristic cluster size vanishes as the repulsive glass melts towards *U*_*c*_, whereas the same measure diverges when *U*_*c*_ is approached from the attractive side (Fig. 5b).

These clusters of fast particles that are characteristic of heterogeneous dynamics in supercooled liquids emerge within the amorphous solid due to subtle differences in local structure. As collective thermal fluctuations continuously randomize the local surroundings of each particle, we may expect these clusters to be transient and dissolve and emerge throughout the solid as time proceeds. We compute the average life time *τ*_*cl*_ that a single particle is connected to a cluster as a proxy for the cluster dynamics. Interestingly, we find a distinctly different behavior in the re-entrant liquid as compared to the glassy phases. In the liquid, the cluster lifetime decays exponentially, with a characteristic decay time 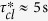, which corresponds roughly to the Brownian time scale of the particles (triangles in Fig. 5c). Simple diffusion thus governs the cluster dynamics in the liquid as expected. However, in the glassy state, the exponential decay gives way to a powerlaw probability distribution (circles and squares in Fig. 5c). This indicates that there is no longer a characteristic time scale for transient cluster dynamics. This suggests that not only the structure of these clusters is fractal^17^ but that their scale-free nature extends to their dynamics. Surprisingly, this metric does not show any difference between the repulsive and attractive glasses. We speculate that the emergence and dissolution of the “fast” clusters is governed by very small and local positional fluctuations of the particles. The short-time dynamics of the colloids in the glass are relatively unaffected by attraction strength, as compared to the long-time diffusive behavior which shows a strong dependence on the particle interactions (Fig. 2).

## Conclusion

These results show how small changes in local structure, signalling the onset of bond formation, give rise to large changes in global and local particle dynamics when a repulsive glass transforms into an attractive glass. The attraction-induced melting of the repulsive glass shows strong similarities to its behavior at *U* = 0 with changing *ϕ*. By contrast, the emergence of the attractive glass is strongly bonding-driven, exhibiting a distinct critical onset as the suspension crosses the gel line. Within the attractive glass dynamics are revealed to be complex, with different modes of relaxation acting simultaneously. Our results highlight the different microscopic physics which govern repulsive and attractive glasses and shed new light on the competition between bonding and caging at the boundary between these two different amorphous solids.

## Methods

### Colloidal glasses

We study a colloidal glass of fluorescent poly(methyl methacrylate) particles, stabilised by poly(hydroxy-stearic acid). The particles are synthesized following established protocols^23^,^24^ and labelled with the dye Nile Red. After cleaning by repeated washing against hexanes, the particles are suspended and equilibrated in an index- and density-matching mixture of cyclohexyl bromide, decaline and tetralin. To ensure hard-sphere like interactions, we add 260 nM tetrabutylammonium bromide to screen residual charge interactions^25^. We use a bidisperse mixture of particles with radii *a*_*A*_ = 0.89 and *a*_*B*_ = 1.23 *μm* radius to avoid crystallisation even after prolonged equilibration. At this size ratio we do not observe decoupling between the diffusivity of the two sizes^19^. Quantifying the exact value of the volume fraction of concentrated suspensions is notoriously difficult especially for bidisperse mixtures^26^; we determine the effective volume fraction at *ϕ* ≈ 0.57 by sedimenting a diluted stock and assuming a random close packed sediment at *ϕ* ≈ 0.65. In all our experiments *ϕ* is kept strictly constant such that errors in determining *ϕ* do not lead to systematic error in our analysis.

Depletion interactions are induced by addition of polystyrene microgels, synthesized as following^27^. In brief: 12 gr of hexadecyltrimethylammonium bromide (CTAB) is dissolved in 138 gr deionized water. In a separate flask, we prepare a mixture of 15 ml styrene, 75 *μl* divinylbenzene and 225 mg 2,2-azobis(2-methylpropionitrile) (AIBN). We subsequently emulsify the styrene mixture in the surfactant solution, aided by high-intensity ultrasonic treatment to create a stable microemulsion. After mixing we purge the reaction mixture with nitrogen and initiate the polymerisation by heating to 65 °C. The reaction is allowed to proceed overnight. The particles are purified by precipitation in cold methanol and redissolution in tetrahydrofuran; we repeat this procedure 3 times to ensure complete removal of impurities. The particles are then dried in vacuo. The resulting polystyrene microgels exhibit a hydrodynamic radius of *a*_*μg*_ = 21 nm in the solvent mixture described above.

### Confocal microscopy

The samples are loaded into hermetically sealed glass sample chambers; after equilibration for at least several hours, images are recorded using a Visitech VT-infinity3 equipped with a Hamamatsu ORCA Flash 4.0 camera. For each sample we acquire 5000 three-dimensional image volumes of 52 × 52 × 22 *μ*m at 2 stacks/s. From these data, particle coordinates are extracted in three-dimensions and time using established routines^28^ with a spatial resolutions of 30 nm in *xy* and 60 nm in *z*.

## Additional Information

**How to cite this article**: Laar, T. *et al*. Discontinuous nature of the repulsive-to-attractive colloidal glass transition. *Sci. Rep.* **6**, 22725; doi: 10.1038/srep22725 (2016).

## References

[b1] Hunter G. L. & Weeks E. R. The physics of the colloidal glass transition. Rep. Prog. Phys. 75, 66501 (2012). 10.1088/0034-4885/75/6/066501 22790649

[b2] Pusey P. N. . Hard spheres: crystallization and glass formation. Phil. Trans. R. Soc. A 367, 4993–5011 (2009). 19933124 10.1098/rsta.2009.0181

[b3] van Megen W. & Underwood S. M. Glass transition in colloidal hard spheres: Mode-coupling theory analysis. Phys. Rev. Lett. 70, 2766–2769 (1993). 10053647 10.1103/PhysRevLett.70.2766

[b4] Eckert T. & Bartsch E. Re-entrant Glass Transition in a Colloid-Polymer Mixture with Depletion Attractions. Phys. Rev. Lett. 89, 125701 (2002). 12225101 10.1103/PhysRevLett.89.125701

[b5] Pham K. N., Egelhaaf S. U., Pusey P. N. & Poon W. C. K. Glasses in hard spheres with short-range attraction. Phys. Rev. E 69, 11503 (2004). 10.1103/PhysRevE.69.011503 14995624

[b6] Dawson K. . Higher-order glass-transition singularities in colloidal systems with attractive interactions. Phys. Rev. E 63, 11401 (2000). 10.1103/PhysRevE.63.011401 11304254

[b7] Pham K. N. . Multiple Glassy States in a Simple Model System. Science 296, 104–106 (2002). 11935020 10.1126/science.1068238

[b8] Zhang Z., Yunker P. J., Habdas P. & Yodh A. G. Cooperative Rearrangement Regions and Dynamical Heterogeneities in Colloidal Glasses with Attractive Versus Repulsive Interactions. Phys. Rev. Lett. 107, 208303 (2011). 22181781 10.1103/PhysRevLett.107.208303

[b9] Pham K. N. . Yielding behavior of repulsion- and attraction-dominated colloidal glasses. J. Rheol. 52, 649–676 (2008).

[b10] Koumakis N. & Petekidis G. Two step yielding in attractive colloids: transition from gels to attractive glasses. Soft Matter 7, 2456–2470 (2011).

[b11] Lu P. J. . Gelation of particles with short-range attraction. Nature 453, 499–503 (2008). 18497820 10.1038/nature06931

[b12] Trappe V., Prasad V., Cipelletti L., Segre P. N. & Weitz D. A. Jamming phase diagram for attractive particles. Nature 411, 772–775 (2001). 11459050 10.1038/35081021

[b13] Bergenholtz J. & Fuchs M. Nonergodicity transitions in colloidal suspensions with attractive interactions. Phys. Rev. E 59, 5706–5715 (1999). 10.1103/physreve.59.5706 11969555

[b14] Brambilla G. . Probing the Equilibrium Dynamics of Colloidal Hard Spheres above the Mode-Coupling Glass Transition. Phys. Rev. Lett. 102, 85703 (2009). 10.1103/PhysRevLett.102.085703 19257755

[b15] Patrick Royall C., Williams S. R., Ohtsuka T. & Tanaka H. Direct observation of a local structural mechanism for dynamic arrest. Nat. Mater. 7, 556–561 (2008). 18568032 10.1038/nmat2219

[b16] Biffi S. . Equilibrium gels of low-valence DNA nanostars: a colloidal model for strong glass formers. Soft Matter 11, 3132–3138 (2015). 25747102 10.1039/c4sm02144d

[b17] Weeks E., Crocker J., Levitt A., Schofield A. & Weitz D. A. Three-Dimensional Direct Imaging of Structural Relaxation Near the Colloidal Glass Transition. Science 287, 627–631 (2000). 10649991 10.1126/science.287.5453.627

[b18] Flenner E. & Szamel G. Fundamental differences between glassy dynamics in two and three dimensions. Nat. Commun. 6, 7392 (2015). 26067877 10.1038/ncomms8392 PMC4490572

[b19] Zaccarelli E., Liddle S. M. & Poon W. C. K. On polydispersity and the hard sphere glass transition. Soft Matter 11, 324–330 (2015). 25412138 10.1039/c4sm02321h

[b20] Yodh A. . Entropically driven self–assembly and interaction in suspension. Phil. Trans. R. Soc. A 359, 921–937 (2001).

[b21] Simeonova N. B. . Devitrification of colloidal glasses in real space. Phys. Rev. E 73, 1–5 (2006). 10.1103/PhysRevE.73.041401 16711794

[b22] Olsen N. B., Christensen T. & Dyre J. C. Time-Temperature Superposition in Viscous Liquids. Phys. Rev. Lett. 86, 1271–1274 (2001). 11178061 10.1103/PhysRevLett.86.1271

[b23] Antl L. . The preparation of poly(methyl methacrylate) latices in non-aqueous media. Colloid and Surfaces 17, 67–78 (1986).

[b24] Elsesser M. T. & Hollingsworth A. D. Revisiting the Synthesis of a Well-Known Comb-Graft Copolymer Stabilizer and Its Application to the Dispersion Polymerization of Poly(methyl methacrylate) in Organic Media. Langmuir 26, 17989–17996 (2010). 21053983 10.1021/la1034917

[b25] Royall C. P., Poon W. C. K. & Weeks E. R. In search of colloidal hard spheres. Soft Matter 9, 17–27 (2013).

[b26] Poon W. C. K., Weeks E. R. & Royall C. P. On measuring colloidal volume fractions. Soft Matter 8, 21–30 (2012).

[b27] Antonietti M. . Synthesis and size control of polystyrene latices via polymerization in microemulsion. Macromolecules 24, 6636–6643 (1991).

[b28] Gao Y. & Kilfoil M. L. Accurate detection and complete tracking of large populations of features in three dimensions. Opt. Express 17, 4685–4704 (2009). 19293898 10.1364/oe.17.004685

